# Genetic Diversity, Molecular Phylogeny, and Selection Evidence of Jinchuan Yak Revealed by Whole-Genome Resequencing

**DOI:** 10.1534/g3.118.300572

**Published:** 2018-01-15

**Authors:** Daoliang Lan, Xianrong Xiong, Tserang-Donko Mipam, Changxiu Fu, Qiang Li, Yi Ai, Dingchao Hou, Zhixin Chai, Jincheng Zhong, Jian Li

**Affiliations:** * Institute of Qinghai-Tibetan Plateau, Southwest University for Nationalities, Chengdu 610041, China; † College of Life Science and Technology, Southwest University for Nationalities, Chengdu 610041, China; ‡ Sichuan Province Head Station for Animal Husbandry and Veterinary Medicine, Chengdu 610041, China; § Jinchuan Animal Husbandry and Veterinary Bureau of Aba Tibetan and Qiang Autonomous Prefecture of Sichuan Province, 624100, China

**Keywords:** Jinchuan yak, genome, genetic diversity, molecular phylogeny, selection evidence

## Abstract

Jinchuan yak, a newly discovered yak breed, not only possesses a large proportion of multi-ribs but also exhibits many good characteristics, such as high meat production, milk yield, and reproductive performance. However, there is limited information about its overall genetic structure, relationship with yaks in other areas, and possible origins and evolutionary processes. In this study, 7,693,689 high-quality single-nucleotide polymorphisms were identified by resequencing the genome of Jinchuan yak. Principal component and population genetic structure analyses showed that Jinchuan yak could be distinguished as an independent population among the domestic yak population. Linkage disequilibrium analysis showed that the decay rate of Jinchuan yak was the lowest of the domestic yak breeds, indicating that the degree of domestication and selection intensity of Jinchuan yak were higher than those of other yak breeds. Combined with archaeological data, we speculated that the origin of domestication of Jinchuan yak was ∼6000 yr ago (4000–10,000 yr ago). The quantitative dynamics of population growth history in Jinchuan yak was similar to that of other breeds of domestic and wild yaks, but was closer to that of the wild yak. No significant gene exchange between Jinchuan and other domestic yaks occurred. Compared with other domestic yaks, Jinchuan yak possessed 339 significantly and positively selected genes, several of which relate to physiological rhythm, histones, and the breed’s excellent production characteristics. Our results provide a basis for the discovery of the evolution, molecular origin, and unique traits of Jinchuan yak.

The yak (*Bos grunniens*), also known as “plateau ship,” is a bovid species mainly distributed in the Qinghai–Tibetan Plateau of China and its adjacent alpine or subalpine regions, such as Pakistan, Nepal, Bhutan, India, and Afghanistan (Lan *et al.* 2014). Yaks are the only bovids that can survive at high altitudes (average altitude of 3000 m). They can adapt well to the alpine grassland environment, live freely, and reproduce under harsh plateau environmental conditions, such as thin air, low temperatures, strong ultraviolet radiation, and short grasses (Miao *et al.* 2015). They provide milk, meat, wool, service force, and fuel for local pastoralists. They are the only animals that can maximize herbage resources on the Qinghai–Tibetan Plateau and have an irreplaceable ecological and economic status in their distribution area. They are also important contributors to the gene pool (Lan *et al.* 2014).

More than 17.6 million yaks exist worldwide, and most of them are distributed in plateau regions, such as Qinghai, Tibet, Sichuan, Gansu, Xinjiang, and Yunnan in China, thereby accounting for 95% of the total number of yaks in the world (Ma *et al.* 2013). Except for wild yaks, those in China are currently classified into 12 identified native breeds of domestic yak, such as Jiulong and Maiwa yaks, according to their appearance, morphological structure, production performance, and geographical location (Song *et al.* 2015). Jinchuan yaks, a unique group of yaks, were recently discovered in valley areas in Jinchuan County of Ngawa Tibetan and Qiang Autonomous Prefecture in Sichuan Province, which is located at 101°40′–101°41′E and 31°32′–31°34′N and at 4200–4600 m altitude. These yaks can be found in the northern part of the Great Snowy Mountains of the Hengduan Mountains, in the southeast part of the Qinghai–Tibet plateau, in the northern part of the Northwest Sichuan Plateau, and between the Dadu River and the Ya-lung River. Their grazing pasture is alpine meadow grassland (Mipam *et al.* 2012).Compared with other breeds of yaks, 52% of Jinchuan yaks have 15 thoracic vertebrae and 15 corresponding pairs of ribs, one additional thoracic vertebra, and one additional pair of ribs (Figure 1). In addition, Jinchuan yaks show good meat and milk production and reproductive performance. They exhibit up to 90% reproductive performance, whereas other yaks achieve 70%. The milk, meat yield, and quality of Jinchuan yaks are superior to those of other regional yaks (Mipam *et al.* 2012). Jinchuan multi-rib yaks are a rare yak germ plasm resource, and their beneficial multi-rib mutation characteristic has a high value for popularization and application.

**Figure 1:**
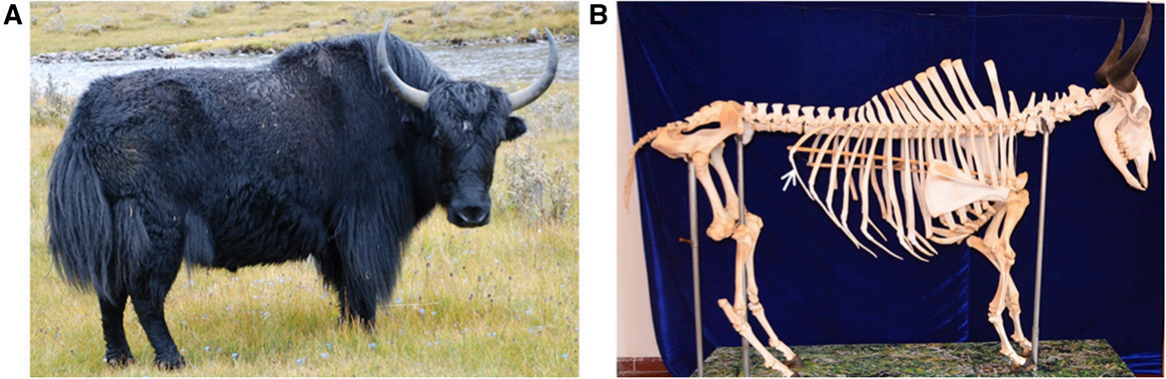
Graphical representation of Jinchuan yaks. (A) Image. (B) Skeleton. Arrows indicate excess ribs.

The mitochondrial haplotype and single nucleotide diversity of Jinchuan yaks are more abundant than those of yaks in other areas (Mipam *et al.* 2012). However, there is limited information about the relationship of the whole genetic structural characteristics, possible origin, and evolution of Jinchuan yaks and those in other regions. The complete yak genome sequence and recent publications on various local yak genomes were the basis for this study (Qiu *et al.* 2012, 2015). Here, the possible origin, evolutionary history, and molecular phylogeny of Jinchuan yaks were determined by resequencing their genome and analyzing published yak genome data. This study aimed to reveal the genetic structural characteristics of Jinchuan yaks at the genome level and provide a basis for further evaluation of their unique molecular formation.

## Materials and Methods

### Sample collection and high-throughput sequencing

**Figure 2:**
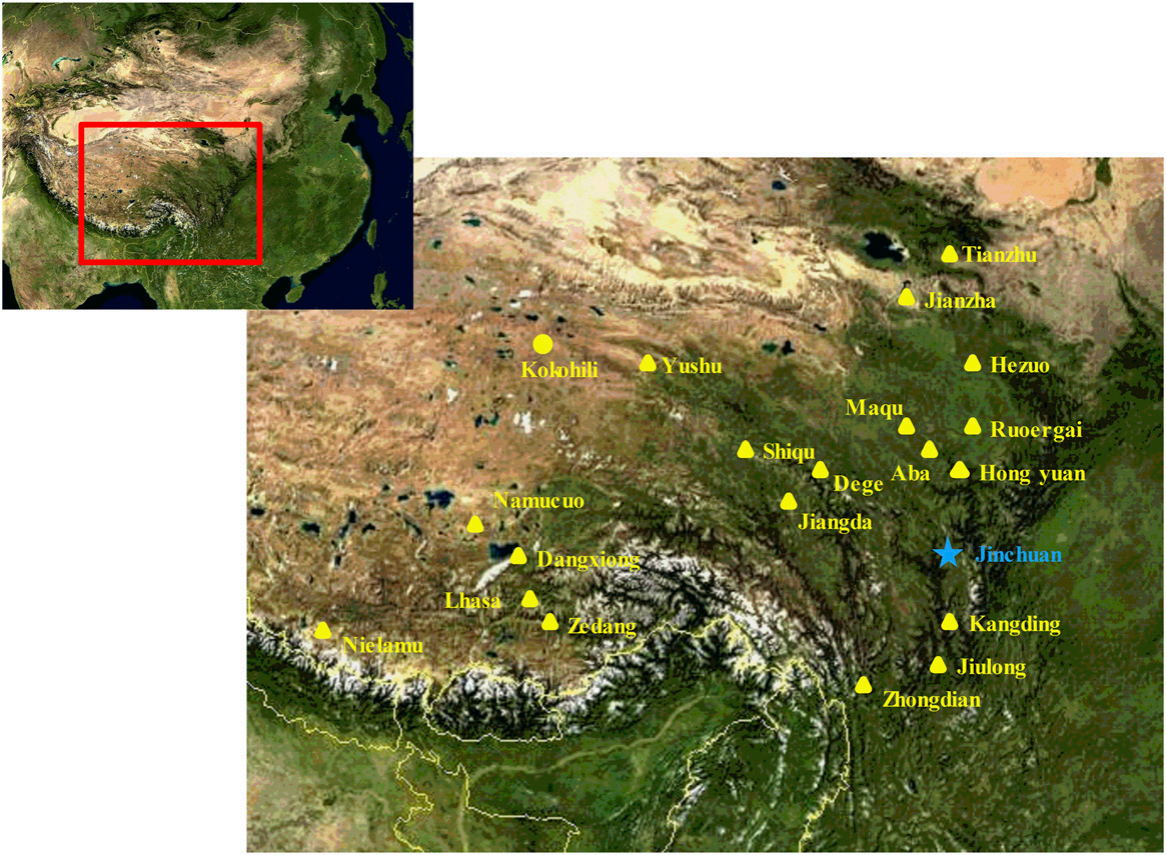
Geographical distribution of the selected yaks in this study. The blue star indicates the location of Jinchuan yaks, triangles correspond to locations of other domestic yaks, and the circle shows the location of wild yaks.

Six female Jinchuan yaks, three with15 ribs and three with 14 ribs, of purely natural grazing and roughly identical health, growth development, and age, were chosen from a group in the yak core zone in Jinchuan County of Ngawa Tibetan and Qiang Autonomous Prefecture of Sichuan Province (Figure 2) for blood sampling. This study was approved by the Animal Ethics and Welfare Association of the Southwest University for Nationalities (No. 16053). Genomic DNA was isolated from blood samples via standard phenol–chloroform extraction. The quality and integrity of the extracted DNA was verified using a Nanodrop ND-1000 spectrophotometer (LabTech) at an absorbance ratio of 260/280 nm and an Agilent 2100 bio-analyzer (Agilent). The paired-end sequencing libraries of each sample with an insert size of 500 bp were constructed according to Illumina’s sequencing instructions on a Hi-Seq 4000 platform (BGI, Shenzhen, China). Certain genomic data (Supplemental Material, Table S1 in File S1) were selected from published local breeding genomic data specific to the geographical location of Jinchuan yaks and compared with those of yak species from other places (Figure 2).

### Sequence quality checking and filtering

To avoid raw reads with artificial bias during library construction and sequencing, or low-quality reads resulting from base-calling duplicates and adapter contamination, we filtered the original Jinchuan yak genome sequence and chose relevant data from local yak breed genome sequences in accordance with the following unified criteria. Reads containing adaptor sequences were filtered. Paired reads were removed when the content of *N* in a single read exceeded 10% of the read length and when single reads contained a low-quality base number (Q ≤ 5) in >50% of the read length. High-quality and clean data were obtained through stringent filtering of the sequencing data.

### Read alignment

After uniform filtering was completed, the obtained high-quality clean reads were mapped to the reference yakgenome1.1 (Hu *et al.* 2012) using the Burrows–Wheeler Aligner (BWA) software (Li and Durbin 2009) with default parameters except for the “-t -k 32 -M -R” option. The BWA software set up a set of stricter parameters to judge alignment and contribute to the increase in the accuracy of single-nucleotide polymorphism (SNP) calling and population variation analyses. The mapping results were converted and indexed to BAM files by utilizing sequence alignment/map (SAM) tools (settings:−bS −t) (Li *et al.* 2009). If multiple read pairs possessed identical external coordinates, the one with the highest mapping quality was retained, and potential PCR duplication was removed to improve the alignment result.

### Variation detection and annotation

After aligning the reads, we performed SNP calling on a population scale using a Bayesian approach implemented in the SAM tool package. The reference genome was subdivided into segments and analyzed in parallel. We then calculated genotype likelihoods from the reads of each individual at each genomic location and allele frequencies in the sample using the Bayesian approach. The “mpileup” command was used to identify SNPs with the parameters “-q 1 -C 50 -t SP -t DP -m 2 -F 0.002.” To exclude SNP calling errors caused by incorrect mapping or InDels, we retained only high-quality SNPs [*i.e.*, coverage depth ≥3, root mean square mapping quality ≥20, miss ≤0.2, minor allele frequency (MAF) ≥0.05] for subsequent analysis. In addition, raw SNPs were further filtered to meet the following criteria. (1) The sum of read depths across all populations was 31,000. (2) The mapping quality score was >20. (3) Multi-nucleotide polymorphisms were ignored. (4) The average Phred scaled base quality of the variant allele was ≥20. (5) The missing variant (miss) from a population scale was detected at ≤2%. (6) The MAF was >0.05.

SNP annotation was performed according to the yak reference genome using the package ANNOVAR (Version: 2013-05-20) (Wang *et al.* 2010). SNPs were categorized on the basis of genome annotation in exonic regions (*i.e.*, SNPs overlapping with a coding exon), intronic regions (*i.e.*, SNPs overlapping with an intron), splicing sites (SNPs within 2 bp of a splicing junction), upstream and downstream regions (SNPs within a 1 kb region upstream or downstream of a transcription start site), and intergenic regions. SNPs in coding exons were further grouped into synonymous SNPs, which do not cause amino acid changes, and nonsynonymous SNPs, which cause amino acid changes. Mutations that induced stop gain and loss were also classified in this group. Only high-quality SNPs were annotated.

### Population genetic polymorphisms

We used all high-quality SNPs of the individuals to infer the population structure. To clarify the phylogenetic relationship from a genome-wide perspective, we constructed an individual-based neighbor-joining tree with 1000 bootstrap values based on p-distance using the TreeBest software (http://treesoft.sourceforge.net/treebest.shtml). We also conducted principal component analysis (PCA) to evaluate the genetic structure by utilizing the GCTA software (http://cnsgenomics.com/software/gcta/mlmassoc.html). The Tracy–Widom test was conducted to determine the significance level of the principal components. After the SNP data were transformed to PLINK files using PLINK (2.00 alpha version) (http://www.cog-genomics.org/plink/2.0/), the population genetic structure was further inferred using ADMIXTURE (https://www.genetics.ucla.edu/software/admixture/). Presumed ancestral populations (K) between two and three were run with 10,000 iterations.

### Linkage disequilibrium (LD) analysis

Squared correlation coefficients (*r*^2^) between pairwise SNPs with MAF > 0.05 were computed using the Haploview software (Barrett *et al.* 2005) to estimate and compare the pattern of LD for different breeds. The parameters in the program were set as “-maxdistance 4 -n -dprime –min MAF 0.05.” The average *r*^2^ was calculated for pairwise markers in a 500 kb window and averaged across the whole genome. We found differences in the rate of decay and level of LD, which reflected variations in population demographic history and effective population size among breeds and populations.

### Gene flow, divergence time estimation, and demographic history analysis

The population relatedness and gene flow patterns between breeding populations were inferred using a maximum likelihood approach implemented in TreeMix (https://bitbucket.org/nygcresearch/treemix/overview). The MCMCTree package in PAML 4.5 (http://abacus.gene.ucl.ac.uk/software/paml.html) was used to estimate divergence times on the basis of the evolutionary tree and to further describe the differentiation time between Jinchuan yak and other groups. We used the putative sequence of a coding region based on the allele frequency of each population, which was concatenated to one supergene for each species. The Bayesian estimate of species divergence times was determined using soft fossil constraints under various molecular clock models. A molecular clock is an effective tool with which to estimate species divergence times. Available information on species divergence times from fossil or geological records can be utilized to calibrate phylogeny and estimate divergence times for all nodes in a tree. We used a pairwise sequentially Markovian coalescent (PSMC) model to infer the demographic history. The history of changes in population size over time was reconstructed by applying the distribution of the most recent common ancestor between two alleles in a sample. The parameters used for the analysis were “-g3 -μ5.84 × 10^−9^-N25 -t15 -r5 -p “4+25∗2+4+6,” similar to those used in a previous yak study (Qiu *et al.* 2015).

### Selective sweep and functional enrichment analyses

To detect the signatures of the selection associated with Jinchuan yak, we evaluated genome-wide variations between Jinchuan yak and other groups. The allele frequencies of variable sites were used to identify regions potentially affected by long-term selection using two complementary approaches. Fixation statistics (FST) and nucleotide diversity (θπ) were calculated with 40 kb sliding windows that had a 20 kb overlap between adjacent windows. A log function was used to Z-transform the distribution of FST and the θπ ratio. Windows at the top 5% of log-odds ratios for FST and θπ were considered as putative selection target regions. The functions of the selected genes were annotated by submitting them to the Gene Ontology (GO) and Kyoto Encyclopedia of Genes and Genomes (KEGG) databases for enrichment analyses. A binomial distribution probability approach corrected by the false discovery rate was used to test significantly enriched gene functions at *P* < 0.05.

### Data availability

The raw data files obtained in this study by Illumina sequencing have been submitted to the Sequence Read Archive Database of the National Center for Biotechnology Information. The accession numbers of sequence read archive runs of six samples are SRR5641605, SRR5641604, SRR5641601, SRR5641603, SRR5641602, and SRR5641600. Genomic data selected from published local breeding genomic data of yaks are available in Table S1 in File S1.

## Results

### Sequencing, detection, and annotation of genome-wide SNPs

Raw sequencing data (400.72G) from six Jinchuan yaks were obtained after sequencing. The yak genome is typically 2.66G, whereas its effective genome data are 2.54G. Genome resequencing yielded an average depth of 25× in each sample. After quality check and filtering were completed, 394.08G high-quality clean reads were obtained for further analysis. After mapping the valid filtered high-quality sequence data to the genome, we observed that the average mapping ratio of Jinchuan yak samples was 98.10%, with an average coverage of 99.14% and sequencing depth of 21.87×. To compare Jinchuan yaks with the population distributed in other regions, we screened the original genome sequencing data of Jinchuan yaks and breeds in other regions on the basis of a unified standard. The available genome data of the yak breeds in other regions showed an average sequencing depth of 6×. Thus, we adjusted the sequencing data of the screened Jinchuan yak sample to obtain an average sequencing depth similar to that of the genome data of yak breeds in other regions. The average mapping ratio of the population sample, sequencing depth of the genome, and coverage were 97.39, 6.77, and 97.66%, respectively.

After filtering, we obtained 7,693,689 valid quality SNPs for further analysis. These SNPs were then annotated. Results (Table S2 in File S1) indicated that SNPs in the intergenic region accounted for a major proportion (76.31%) and the inclusive area corresponded to 21.73%. By comparison, the exon area was merely 0.62%, which comprised 24,812 synonymous mutations and 22,418 nonsynonymous mutations. Furthermore, the ratio of transition mutations (5,539,543) to transversion mutations (2,154,146) was 2.57.

### Population genetic polymorphism and LD decay

**Figure 3:**
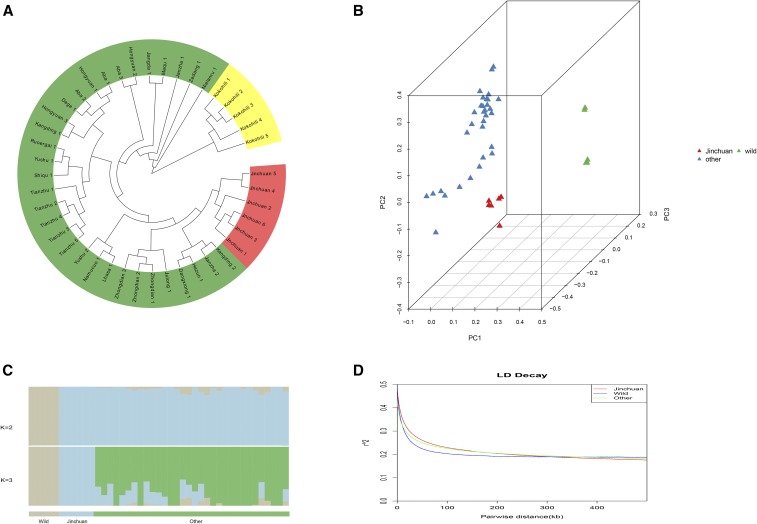
Population genetic polymorphism and LD decay analysis of Jinchuan yak. (A) Phylogenetic tree analysis. Jinchuan yaks are indicated in red, other domesticated yak groups are denoted in green, and wild yaks are shown in yellow. (B) PCA. (C) Population genetic structure analysis. (D) LD decay analysis.

To examine the genetic population structure and relationships between Jinchuan yaks and other yak groups, we conducted a genome-wide population genetic polymorphism analysis. The neighbor-joining tree based on genome-wide data revealed that the yak population in China is largely divided into wild and domesticated yaks. Of these categories, Jinchuan yak is an independent branch and has a close genetic evolutionary relationship with certain yaks in the Kangding, Jianzha, Dangxiong, and Hezuo districts (Figure 3). Using the first, second, and third eigenvectors, we divided the whole yak samples of the population into three groups through PCA: wild, Jinchuan, and other yak breeds. Further population genetic structural analysis indicated that the yak population in China can be classified into wild and other yaks at *K* = 2. At *K* = 3, one genetic structural population of Jinchuan yak, along with wild yak, was obtained, indicating that Jinchuan yak exhibits uniqueness compared with other domestic yaks. To estimate the LD patterns in various yak groups, we calculated the squared correlations of allele frequencies against the genome distance (*r*^2^) between pairs of SNPs. As shown in Figure 3, the fastest attenuation was observed in wild yaks, followed by other yak breeds. Conversely, the slowest attenuation was found in Jinchuan yaks. Thus, the degree of domestication and selection intensity of Jinchuan yaks were greater than those of other yak populations.

### Gene flow, divergence time, and demographic history analysis

**Figure 4:**
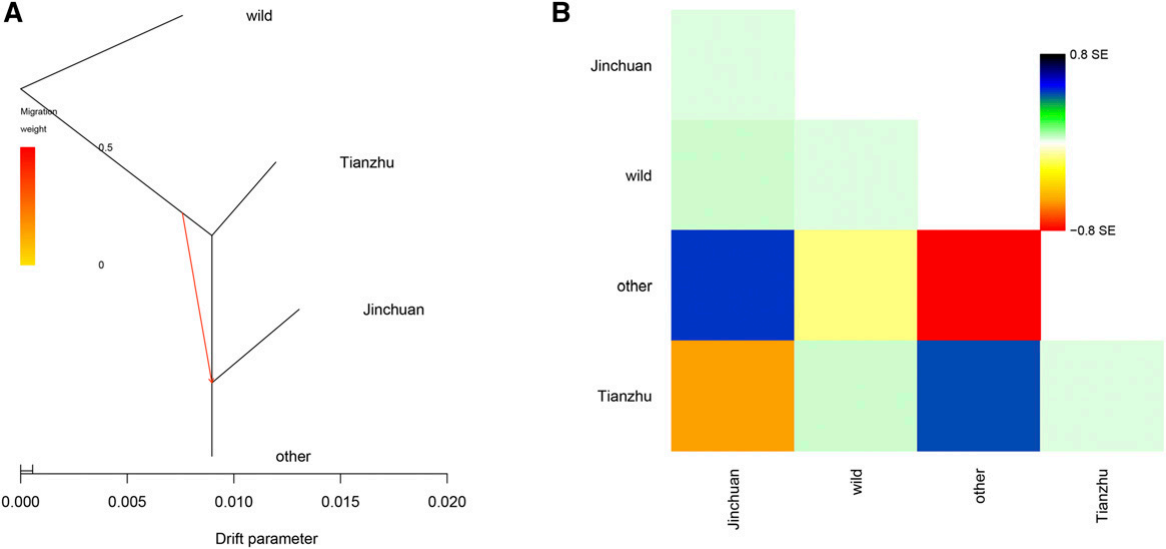
Gene flow analysis of Jinchuan yak. (A) Maximum likelihood tree with two migration events. The arrows (migration events) are colored according to their weight. The horizontal branch length is proportional to the degree of genetic drift in the branch. The scale bar on the left shows 10 times the average SE of the entries in the sample covariance matrix. (B) Residual fit from the maximum likelihood tree in (A).

**Figure 5:**
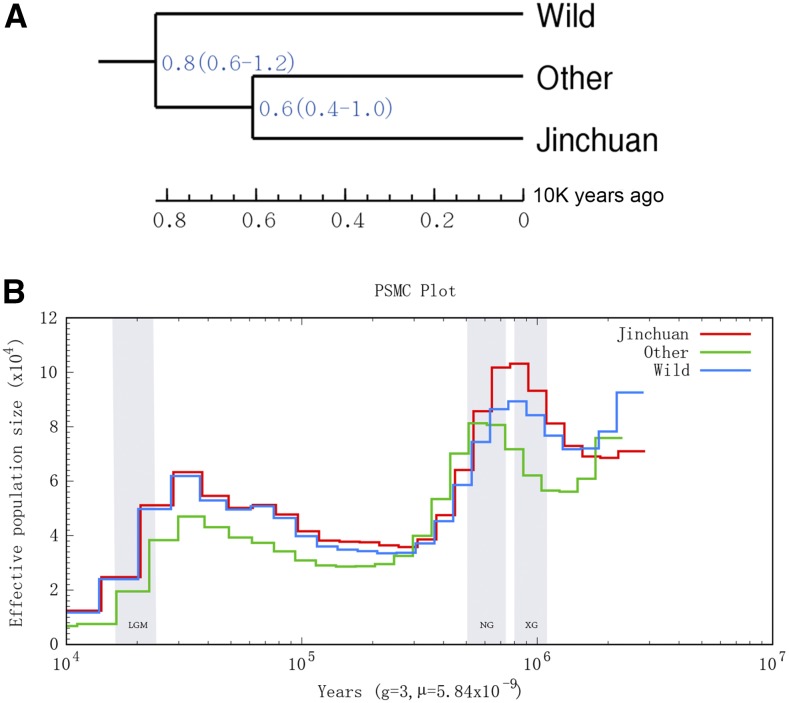
Differentiation time and demographic history of Jinchuan yak. (A) Differentiation time analysis. (B) Demographic history inferred by PSMC for different populations. Changes in the effective population size of the ancestral population of three yak populations, namely Jinchuan, other domesticated yaks, and wild yaks. Time is plotted along the *x*-axis, and the effective population size is represented along the *y*-axis. Colored lines indicate various populations. XG (Xixiabangma Glaciation; 1170–800 thousand years ago, kya), NG (Naynayxungla Glaciation; 780–500 kya), and LGM (last glacial maximum; ∼20 kya) are shaded in gray.

The bioinformatic analysis of gene flow (Figure 4) indicated no obvious genetic communication between wild and domesticated yaks, or domesticated and all yak populations. Interestingly, a yak gene flowed from the ancestor to the descendant population of Jinchuan yaks and other yak populations. Combined with the available literature and fossil archaeological data, the differentiation time of Jinchuan yak was analyzed (Figure 5A). Results demonstrated that the earliest differentiation took place in wild yaks. The differentiation of Jinchuan and other yaks occurred ∼6000 yr (4000–10000) ago. PSMC analysis indicated that the development track of the quantitative kinetics of Jinchuan yak population resembled that of wild yaks and other domesticated yak breeds but exhibited a greater similarity to wild yaks than to other breeds. The effective ancestor population size of Jinchuan yak showed two peaks. One peak appeared during 96.9 thousand and 600 thousand years ago (kya) in the later Xixiabangma Glaciation period. Afterward, the population size decreased considerably. Its size satisfied the second peak during 62K and 30 kya in the early last glacial maximum before this size subsequently decreased (Figure 5B).

### Genome-wide selective sweep signals and functional analysis

**Figure 6:**
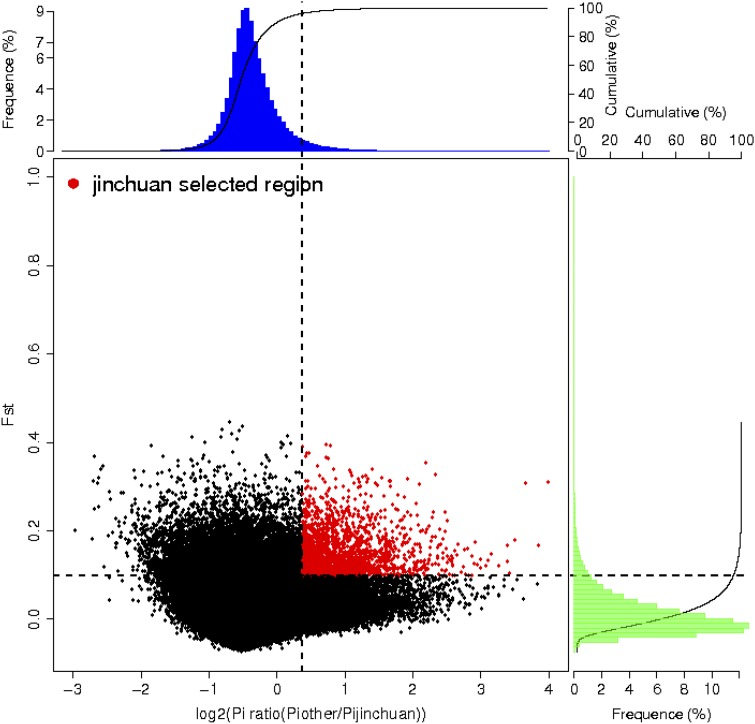
Genome-wide selective sweep analysis of Jinchuan yak. Distribution of θπ ratio (θπ, other domesticated yaks/θπ, Jinchuan yak) and FST values calculated in 500 kb windows sliding in 10 kb steps. Data points in red are regions under selection in Jinchuan yaks.

We measured genome-wide variations between the Jinchuan and other domesticated yak groups to detect the signatures of selection associated with the uniqueness of Jinchuan yak. Based on the results, 339 genes of the Jinchuan yak population were selected (Figure 6). The selected genes were further subjected to functional analysis. Among the top 10 enriched categories in GO functional analysis (Table S3 in File S1), the GO category related to physiological rhythm accounted for 50% (5/10), followed by two GO categories associated with carbohydrate metabolism (2/10). KEGG analysis revealed the involvement of these genes in many pathways. Among the top 10 pathways (Table 1), lysine degradation, systemic lupus erythematosus, and alcoholism displayed the greatest enrichments, followed by adipocytokine signaling and cardiac muscle contraction pathways. In addition to these pathways, several pathways linked with the special traits of Jinchuan yaks were discovered (Table 2). These pathways included those related to skeletal development, such as dorso-ventral axis formation and osteoclast differentiation, and those associated with reproduction, such as oocyte meiosis and oxytocin signaling.

**Table 1:** Top 10 enriched KEGG pathways in the selected genes

	Pathway	Genes Involved in Pathway	*P*-Value
1	Lysine degradation	LONP2,IQCk, ATPBD4,PPP1R1C,ARIH1,AAED1	8.30E−06
2	Alcoholism	H2A.1, H2AFJ,H4, H3.1,CAMKK2, H2B.1B	0.000569
3	Systemic lupus erythematosus	H2A.1, H2AFJ,H4, H3.1,H2B.1B	0.000907
4	Cytosolic DNA-sensing pathway	POLR2F,STING,IL33	0.015158
5	Adipocytokine signaling pathway	IRS2,STK11, CAMKK2	0.021361
6	Cardiac muscle contraction	UQCR11,OK/SW-CL.16,MYO7A	0.027874
7	Parkinson’s disease	UQCR11,OK/SW-CL.16,PINK1, MYO7A	0.037931
8	Pyrimidine metabolism	POLR2F, TS, NME5	0.051757
9	Sphingolipid metabolism	UGT8, PLPP3	0.060316
10	Huntington’s disease	POLR2F, UQCR11,OK/SW-CL.16, MYO7A	0.074228

**Table 2:** Enriched KEGG pathways related to the peculiarity of Jinchuan yaks in the selected genes

	Pathway	Genes Involved in Pathway
1	Dorso-ventral axis formation	ETV6
2	Osteoclast differentiation	Mitf, Ifnar1
3	Oocyte meiosis	CDC23, PTTG1
4	Oxytocin signaling pathway	CAMKK2

## Discussion

Jinchuan yak is a newly discovered yak breed characterized by the presence of multiple ribs and other excellent characteristics, such as high meat and milk yield and strong reproductive performance, compared with other domesticated yak groups (Mipam *et al.* 2012). Thus, this breed is of high promotional and application value. However, there is limited information about its overall genetic structure, relationship with yaks in other regions, and origin and evolutionary processes. Considering this limitation, we conducted an in-depth genome resequencing by comparing the published and analyzed yak data. We further enriched the background information and evaluated the uniqueness of Jinchuan yak.

Genetic evolution analysis revealed that Chinese yaks are mainly divided into two large groups. One group consists of wild yaks, whereas the other group comprises domesticated yaks. Consistent with the result of Qiu *et al.* (2015), we found no evident distinction between domesticated yaks in various regions. Jinchuan yaks occurred as a small independent branch and demonstrated a close genetic relationship with a few of the yaks in Kangding, Jianzha, Dangxiong, and Hezuo. Further PCA and group genetic structure analysis confirmed that wild yaks belong to one group, whereas domesticated yaks constitute another group. With the increase and detailed division of the *K* value, Jinchuan yaks have separated and formed an independent group. Gene flow analysis showed no evident genetic exchange between wild and domesticated yaks, or among various groups of domesticated yaks. Interestingly, a branch of a yak ancestor leading to Jinchuan yak was observed, suggesting that Jinchuan yak is a unique breed among other domesticated yaks. The LD analysis results illustrated that Jinchuan yak yielded the lowest attenuation speed among domesticated yaks, implying that the degree of domestication and selection intensity of Jinchuan yaks were higher than those of other yaks. These traits could, therefore, account for the uniqueness of Jinchuan yaks. In terms of the origin of Jinchuan yak, documents and fossil archaeological data (Cai 1991; Cai and Gerald 1995) combined with genome information indicated that the differentiation time of Jinchuan yak was ∼6000 yr (ranging from 4000 to 10,000) ago. That is, Jinchuan yaks were domesticated in this period. Consistent with previous results (Qiu *et al.* 2015), PSMC analysis revealed that the dynamic development trajectories of the domesticated and wild yak populations were similar. However, the dynamic development trajectory of the Jinchuan yak population was more similar to that of wild yaks than other groups of yaks. The effective population size of the ancestor of Jinchuan yak exhibited two expansions. However, both expansions decreased sharply in relation to the glacial period. In addition, the populations of other species, such as panda, golden monkey, and other organisms on the Qinghai–Tibet plateau (Zhao *et al.* 2013; Zhou *et al.* 2014), decreased in the same period. In the glacial period, the environment of the Qinghai–Tibet plateau likely underwent remarkable changes, thereby causing a decline in the populations of species.

Whole-genome selection analysis revealed that 339 genes of Jinchuan yaks were significantly and positively selected compared with those of other yak species. Further functional analysis on these positively selected genes indicated that the circadian rhythm-related GO categories, such as the LDLRAD3, ATPBD4, DPH6, Syn2, cSMD3, and MYO3B genes, hold the largest portion among the top 10 GO enrichments. Circadian rhythm-related biological clocks exist in a wide range of organisms. For example, these clocks regulate the behavioral and physiological functions associated with multiple aspects—including sleeping, feeding, and metabolism—in mammals to synchronize the internal physiological rhythm and variation period between an individual organism and the surrounding environment. Thus, evolutionary advantages are obtained after effective acclimatization is achieved (Bass 2012). Among the genes involved in these categories, three were correlated with the nervous system. Syn2 and CSMD3 were associated with the development of synapses (Inoue *et al.* 2012; Mizukami *et al.* 2016), whereas LDLRAD3 was involved in the regulation of a myeloid precursor protein transporter in neuronal cells (Ranganathan *et al.* 2011).The positive selection of brain and nerve-related genes and functional pathways had a vital role in the early stages of rabbit and cattle domestication (Carneiro *et al.* 2014; Montague *et al.* 2014). Research on yaks has also confirmed that genes related to neural development and behavior participate in yak domestication (Qiu *et al.* 2015). This study verified that neural rhythm-related genes were positively selected in Jinchuan yaks but were different from the types of nerve-related genes found in a previous study on yaks. Nevertheless, the specific function of the former should be further examined. In addition to the circadian rhythm-related GO categories, arabinose metabolism-related GO categories, such as the GNRHR1, HYDIN, SLC6A13, IL-33, and PPP1R1C genes, occupied a large proportion of the top 10 GO enrichment classifications. Among these genes, SLC6A13 is implicated in the transport of amino acids in synapses (Zhou *et al.* 2012), further confirming that nerve-related genes are positively selected in Jinchuan yaks.

Further KEGG analysis indicated that lysine degradation, systemic lupus erythematosus, and alcoholism were the most significantly enriched among the top 20 pathways. The selected genes related to the systemic lupus erythematosus and alcoholism pathways were basically the same and were mainly composed of histone genes, such as H2A.1, H2A.J, H4, H3.1, and H2B.1B. Histones are basic proteins present in the chromatin of eukaryotic cells. As major components of the nucleosome, histones play an important part in genome transcription regulation, DNA repair and replication, and chromosome stability. Moreover, histones can be subjected to various modifications, such as methylation, acetylation, phosphorylation, ADP-ribosylation, and ubiquitination. Such modifications have important functions in various physiological activities, particularly the epigenetic regulation of genes (Rothbart and Strahl 2014).The significantly positive selection of histone genes in this study suggested that these histone genes may participate in the regulation of multiple spine traits.

In addition to the previously stated top pathways, certain pathways associated with the special traits of Jinchuan yaks were enriched. Examples included pathways related to skeletal development (*e.g.*, dorso-ventral axis formation and osteoclast differentiation) and reproduction (*e.g.*, oocyte meiosis, oxytocin signaling pathway). The two skeletal development-related pathways involved three genes, namely, ETV6, Mitf, and Ifnar1. ETV6 and Mitf are transcription-regulating factors, whereas ETV6 is a transcription-repressing factor implicated in hematopoiesis and malignant transformation (Zhang *et al.* 2015). Mitf regulates the expression of genes in differentiation and proliferation of various cell types (Pogenberg *et al.* 2012). Ifnar1 is a component of the type I interferon receptor, which triggers a series of downstream physiological responses by activating the JAK–STAT cascade via binding to type I interferon, such as a combined transcription with the Mitf gene to regulate the transcription and replication of specific genes of osteoclasts (Odkhuu *et al.* 2012). The two reproduction-related pathways mainly comprise CDC23, PTTG1, and CAMKK2 genes. CDC23 and PTTG1 participate in oocyte maturation, whereas CAMKK2 is involved in oxytocin-related signaling pathways. The sexual maturity of yaks is slow. Additionally, their reproductive capacity is generally lower than those of ordinary cattle living in plain areas. The average reproductive rate of an adult yak is only 48.61%, and half of the yaks give birth every 2 or 3 yr. Moreover, the rate of estrus in female yaks is quite low. Over 90% postpartum female yaks are unable to undergo a period of estrus in the same year (Lan *et al.* 2016). Compared with yaks in other areas, Jinchuan yaks yield reproductive rates of 90% or above. The majority of them can deliver a young yak each year. This study found that the genes involved in oocyte maturation and oxytocin-related pathways were positively selected and, thus, probably provided a basis for further analysis of the excellent reproductive traits of Jinchuan yaks. In addition to characteristics such as multiple ribs and high fecundity, the meat quality of Jinchuan yaks is superior to that of yaks in other areas. The diameter of the muscle fibers of the former is smaller than that of other breeds, with extra delicate meat (Ai *et al.* 2013).Among the top pathways, the muscle-related pathway (cardiac muscle contraction) is composed of three genes, namely UQCR11, OK/SW-cl.16, and MYO7A. MYO7A is a member of the encoding myosin gene family, whose encoding product myosin-VII is not only a myofibril component but also a major muscle protein (Heissler and Manstein 2012). Hence, this member has an important role in muscle movement. Its size determines the size of muscle fibers to a great extent. Pathway-related genes that were positively selected may provide a basis for further analysis of the meat quality of Jinchuan yaks.

In summary, the possible origin, evolutionary history, molecular phylogeny, and selection evidence of Jinchuan yaks were explored in this study. The results provided a basis for further enrichment of the background information of these yaks and evaluated their uniqueness.

## Supplementary Material

Supplemental material is available online at www.g3journal.org/lookup/suppl/doi:10.1534/g3.118.300572/-/DC1.
