# Supplementary material for: Genetic Diversity, Molecular Phylogeny, and Selection Evidence of Jinchuan Yak Revealed by Whole-Genome Resequencing
Source: G3 (Bethesda). 2018 Jan 15;8(3):945–52. doi: 10.1534/g3.118.300572 (PMC5844314; doi:10.1534/g3.118.300572)
Supplement: Supplementary file 1 [file 945FileS1.docx]

| Sample | SRR | Clean_reads | Mapped_reads | Mapping_rate | Average_depth |
| --- | --- | --- | --- | --- | --- |
| Aba_1 | SRR2059901 | 174880808 | 166779816 | 95.37% | 5.95 |
| Aba_2 | SRR2059897 | 237589448 | 232524873 | 97.87% | 8.44 |
| Aba_3 | SRR2059898 | 2.13E+08 | 2.08E+08 | 97.50% | 7.46 |
| Dangxiong_1 | SRR2059935 | 2.42E+08 | 2.35E+08 | 97.01% | 7.41 |
| Dege_1 | SRR2059902 | 1.99E+08 | 1.93E+08 | 97.08% | 6.76 |
| Hezuo_1 | SRR2058187 | 1.7E+08 | 1.65E+08 | 97.24% | 4.94 |
| Hongyuan_1 | SRR2059907 | 1.88E+08 | 1.81E+08 | 96.44% | 6.66 |
| Hongyuan_2 | SRR2059915 | 2.37E+08 | 2.31E+08 | 97.30% | 7.67 |
| Hongyuan_3 | SRR2059908 | 1.92E+08 | 1.88E+08 | 97.87% | 6.71 |
| Jiangda_1 | SRR2059936 | 2E+08 | 1.94E+08 | 97.09% | 6.9 |
| Jianzha_1 | SRR2059875 | 2.09E+08 | 2.03E+08 | 96.85% | 6.96 |
| Jianzha_2 | SRR2059878 | 2.33E+08 | 2.27E+08 | 97.37% | 7.26 |
| Jiulong_1 | SRR2059924 | 1.52E+08 | 1.47E+08 | 97.25% | 5.16 |
| Kangding_1 | SRR2059931 | 2.01E+08 | 1.96E+08 | 97.59% | 6.82 |
| Kangding_2 | SRR2059930 | 1.53E+08 | 1.49E+08 | 97.31% | 5.43 |
| Kokohili_1 | SRR2059962 | 2.84E+08 | 2.77E+08 | 97.30% | 8.74 |
| Kokohili_3 | SRR2059965 | 2.58E+08 | 2.5E+08 | 97.01% | 7.73 |
| Kokohili_4 | SRR2059966 | 2.2E+08 | 2.1E+08 | 95.55% | 6.92 |
| Kokohili_5 | SRR2062306 | 2.05E+08 | 2.02E+08 | 98.60% | 7.57 |
| Lhasa_1 | SRR2059938 | 1.97E+08 | 1.9E+08 | 96.52% | 5.81 |
| Maqu_1 | SRR2058327 | 1.99E+08 | 1.94E+08 | 97.58% | 6.93 |
| Nielamu_1 | SRR2059941 | 2.07E+08 | 2E+08 | 96.73% | 6.95 |
| Ruoergai_2 | SRR2059933 | 1.83E+08 | 1.75E+08 | 95.60% | 6.44 |
| Shiqu_1 | SRR2059943 | 2E+08 | 1.94E+08 | 97.22% | 7.11 |
| Tianzhu_1 | SRR2058047 | 1.97E+08 | 2.02E+08 | 97.65 | 6.93 |
| Tianzhu_2 | SRR2058052 | 2.07E+08 | 2.02E+08 | 97.48% | 6.91 |
| Tianzhu_3 | SRR2058053 | 1.93E+08 | 1.88E+08 | 97.08% | 6.53 |
| Tianzhu_4 | SRR2058055 | 1.75E+08 | 1.72E+08 | 98.10% | 6.25 |
| Tianzhu_5 | SRR2058185 | 1.93E+08 | 1.89E+08 | 97.47% | 6.78 |
| Yushu_1 | SRR2059894 | 2.25E+08 | 2.16E+08 | 96.00% | 7.46 |
| Yushu_2 | SRR2059895 | 2.13E+08 | 2.06E+08 | 96.38% | 6.25 |
| Zedang_1 | SRR2059946 | 2.08E+08 | 2.02E+08 | 97.38% | 7.12 |
| Namucuo 1 | SRR2059939 | 1.73E+08 | 1.79E+08 | 96.83% | 5.47 |
| Zhongdian_1 | SRR6162827 | 91241998 | 89395766 | 97.98% | 4.06 |
| Zhongdian_2 | SRR6162828 | 91394876 | 89008788 | 97.39% | 3.98 |
| Zhongdian_3 | SRR6162829 | 92886414 | 91160037 | 98.14% | 4.15 |

**Additional file1.Table S1. Samples were selected from published yak breeding genomic data**

**Additional file 2.** **Table S2. Category Number of SNPs**

| Category |  | Number of SNPs |
| --- | --- | --- |
| Upstream |  | 49,159 |
|  | Stop gain | 398 |
| Exonic | Stop loss | 23 |
|  | Synonymous | 24,812 |
|  | Non-synonymous | 22,418 |
| Intronic |  | 1,672,011 |
| Splicing |  | 150 |
| Downstream |  | 53,031 |
| Upstream/Downstream |  | 654 |
| Intergenic |  | 5,871,033 |
| ts |  | 5,539,543 |
| tv |  | 2,154,146 |
| ts/tv |  | 2.571 |
| Total |  | 7,693,689 |
|  |  |  |

**Additional file 3. Table S3. Top 10 enrichment GO categories for selected genes**

| No | | GO_accession | Description | pValue | DEG | Gene_names |
| --- | --- | --- | --- | --- | --- | --- |
| 1 | GO:0019566 | arabinose metabolic process | 0.0009157 | 5 | HYDIN, GNRHR,IL-33, GABT2, PPP1R1C |  |
| 2 | GO:0046373 | L-arabinose metabolic process | 0.000916 | 5 | GNRHR, HYDIN, GABT2, IL-33, PPP1R1C |  |
| 3 | GO:0007622 | rhythmic behavior | 0.001349 | 5 | MYO7A, CSMD3, Syn2, [ATPBD4](http://www.baidu.com/link?url=5vTqK-A2hq0ucbol5ZvUE3CcHFKqOmBzyg2Qf3t_4ihlVpkHXO_flpg96C6ItN3ECYFveA3As0-NCHU0dYteT_), LDLRAD3 |  |
| 4 | GO:0022410 | circadian sleep/wake cycle process | 0.001349 | 5 | LDLRAD3,[ATPBD4](http://www.baidu.com/link?url=5vTqK-A2hq0ucbol5ZvUE3CcHFKqOmBzyg2Qf3t_4ihlVpkHXO_flpg96C6ItN3ECYFveA3As0-NCHU0dYteT_), Syn2，CSMD3, MYO7A |  |
| 5 | GO:0042745 | circadian sleep/wake cycle | 0.001349 | 5 | LDLRAD3,[ATPBD4](http://www.baidu.com/link?url=5vTqK-A2hq0ucbol5ZvUE3CcHFKqOmBzyg2Qf3t_4ihlVpkHXO_flpg96C6ItN3ECYFveA3As0-NCHU0dYteT_), Syn2，CSMD3, MYO7A |  |
| 6 | GO:0048512 | circadian behavior | 0.001349 | 5 | LDLRAD3, CSMD3, MYO7A，[ATPBD4](http://www.baidu.com/link?url=5vTqK-A2hq0ucbol5ZvUE3CcHFKqOmBzyg2Qf3t_4ihlVpkHXO_flpg96C6ItN3ECYFveA3As0-NCHU0dYteT_), Syn2 |  |
| 7 | GO:0019321 | pentose metabolic process | 0.001431 | 5 | HYDIN, GNRHR,IL-33, GABT2, PPP1R1C |  |
| 8 | GO:0007623 | circadian rhythm | 0.002549 | 6 | CWC27，LDLRAD3, Syn2，[ATPBD4](http://www.baidu.com/link?url=5vTqK-A2hq0ucbol5ZvUE3CcHFKqOmBzyg2Qf3t_4ihlVpkHXO_flpg96C6ItN3ECYFveA3As0-NCHU0dYteT_), CSMD3, MYO7A |  |
| 9 | GO:0006298 | mismatch repair | 0.003389 | 12 | Ryr2，Grip1，KIAA0753，DGKI，ROBO1, QRFPR, CSPG4, OPHN1, NCOA2, Teneurin-2, MATR3, NWD2 |  |
| 10 | GO:0032886 | regulation of microtubule-based process | 0.004769 | 6 | Txnrd2, EDIL3, CYP2C31,Cntnap4, VPS13D , PDE1A |  |
